# Renoprotective effect of berberine *via* regulating the PGE_2_‐EP1‐Gαq‐Ca^2+^ signalling pathway in glomerular mesangial cells of diabetic rats

**DOI:** 10.1111/jcmm.12837

**Published:** 2016-04-21

**Authors:** Wei‐Jian Ni, Li‐Qin Tang, Hong Zhou, Hai‐Hua Ding, Yuan‐Ye Qiu

**Affiliations:** ^1^ Affiliated Anhui Provincial HospitalAnhui Medical UniversityAnhui ProvinceChina; ^2^ West Branch of Anhui Provincial HospitalAnhui Provincial Cancer HospitalAnhui ProvinceChina

**Keywords:** berberine, diabetic nephropathy, glomerular mesangial cell, signalling pathway, EP1 receptor

## Abstract

G‐protein coupled receptor‐mediated pathogenesis is of great importance in the development of diabetic complications, but the detailed mechanisms have not yet been clarified. Therefore, we aimed to explore the roles of the prostaglandin E2 receptor 1 (EP1)‐mediated signalling pathway and develop a corresponding treatment for diabetic nephropathy (DN). To create the DN model, rats fed a high‐fat and high‐glucose diet were injected with a single dose of streptozotocin (35 mg/kg, i.p.). Then, rats were either treated or not with berberine (100 mg/kg per day, i.g., 8 weeks). Cells were isolated from the renal cortex and cultured in high‐sugar medium with 20% foetal bovine serum. Prostaglandin E_2_ (PGE_2_) levels were determined by ELISA, and cells were identified by fluorescence immunoassay. We measured the biochemical characteristics and observed morphological changes by periodic‐acid‐Schiff staining. The expression of the EP1 receptor and the roles of GRK2 and β‐arrestin2 were identified using western blotting and flow cytometry. Downstream proteins were detected by western blot, while molecular changes were assessed by ELISA and laser confocal scanning microscopy. Berberine not only improved the majority of biochemical and renal functional parameters but also improved the histopathological alterations. A significant increase in PGE_2_ level, EP1 membrane expression and Gαq expression, and concentration of Ca^2+^ were observed, accompanied by increased GRK2 and β‐arrestin2 levels soon afterwards. Berberine decreased the abnormal concentration of Ca^2+^, the increased levels of PGE_2_, the high expression of EP1 and Gαq and suppressed the proliferation of mesangial cells. The EP1 receptor, a critical therapeutic target of the signalling pathway, contributed to mesangial cell abnormalities, which are linked to renal injury in DN. The observed renoprotective effects of berberine *via* regulating the PGE_2_‐EP1‐Gαq‐Ca^2+^ signalling pathway indicating that berberine could be a promising anti‐DN medicine in the future.

## Introduction

Diabetic nephropathy (DN) is believed to be the major cause of morbidity and mortality in patients with diabetes and is highly prevalent in end‐stage renal disease 1. It is characterized by complete or partial microalbuminuria, glomerular mesangial cell (GMC) proliferation with deposition of extracellular matrix (ECM) at the glomerular level, global glomerular sclerosis and atrophy, which ultimately result in progressive renal failure 2, 3. Hyperglycaemia is a necessary precondition in the process of DN, whereas dyslipidaemia is an equally important aggravating factor 4. Many factors, including systemic hypertension with hyperfiltration 5, increased advanced glycation end products (AGEs), protein kinase C (PKC) pathway activation and cytokines, have been identified as critical deteriorating factors that accelerate DN based on genetic susceptibility 6. However, the precise mechanisms are not yet fully elucidated. Prostaglandins are derived from the 20‐carbon chain fatty acid, arachidonic acid (AA) stored in the plasma membrane of cells 7. Prostanoids, including prostaglandins (PGs) PGE2, PGF2a, PGI2, PGD2 and thromboxane A2 (TXA2), are the oldest members of the eicosanoid family. Prostanoids can be biosynthesized from three related fatty acid precursors, 8,11,14‐eicosatrienoic acid (homo‐linolenic acid), 5,8,11,14‐eicosatetraenoic acid (EAA) and 5,8,11,14,17‐eicosapentaenoic acid (EPA or timodonic acid) 8. Once synthesized, prostanoids are transported into the extracellular microenvironment by specific multidrug resistance‐associated proteins (MRPs). After being exported to the microenvironment, prostanoids bind to G‐protein‐coupled receptors that contain seven transmembrane‐spanning domains 9. Among the numerous prostanoids, PGE_2_ has been shown to play significant roles in regulating renal physiology, including glomerular filtration, renin release, tubular salt and water metabolism 10. Multiple actions of PGE_2_ were found to be mediated by specific G‐protein coupled receptors (GPCRs) 11, and local production of PGE_2_ has been shown to be associated with glomerular inflammation and renal injury 12. In glomerular microcirculation, E prostanoid receptor (EP) is one of the most abundant GPCRs, when coupled to the corresponding G protein, regulates renal functional alterations 13. One study showed that EP1, an important EP receptor subtype, plays important roles in diabetic progression, together with PGE_2,_ as suggested by a reduced glomerular size in EP1‐deficient mice 14. Meanwhile, pathophysiological disruptions by the PGE_2_/EP1 axis suggest that selective blockade of EP1 may be a useful therapeutic option in DN 15. Therefore, research on the PGE_2_‐EP1‐related signalling pathway in diabetic rats is urgently needed. Berberine (BBR; [C_20_H_18_NO_4_]^+^), a type of isoquinoline alkaloid, is the major constituent of *Rhizoma coptidis* 16. Research demonstrated that BBR exhibited various pharmacological activities, including antioxidation, anti‐inflammatory, antilipemic and hypoglycaemic activities, which indicated potential clinical and research value as a future therapeutic anti‐DN drug 17. Most recently, research showed that BBR could effectively reduce the PGE_2_ levels in various diseases, which suggested that there might be a relationship between BBR and the PGE_2_/EP axis in the process of DN 18. However, to date, the precise mechanisms are still being investigated. This study aimed to explore the renoprotective effects of BBR and the detailed mechanism *via* the PGE_2_‐EP1‐Gαq‐Ca^2+^ signalling pathway in GMCs of diabetic rats.

## Materials and methods

### Materials

Streptozocin (STZ, S0130) was obtained from Sigma Chemical Co. (St. Louis, MO, USA). Berberine and enalapril were kindly provided by the Anhui Provincial Hospital. Rabbit anti‐EP1 (H‐60, sc‐20674), anti‐GRK2 (C‐15, sc‐562), mouse anti‐Gαq (10, sc‐136181) and anti‐β‐arrestin2 (B‐4, sc‐365445) antibodies were purchased from Santa Cruz Biotechnology, Inc. (Santa Cruz, CA, USA). The Ca^2+^ Fluo‐3, AM (S21010) was purchased from Solarbio Biotechnology, Inc. (Solarbio, Beijing, China). PGE_2_ (14010) and the PGE_2_ enzyme immunoassay kit (514010) were obtained from Cayman Company (Cayman Chemical, Ann Arbor, Michigan, USA). Other chemicals were analytical grade and were purchased from commercial sources.

### Experimental protocol

Male Sprague–Dawley rats (180 ± 20 g. Grade SPF, No. 34000200000613) were obtained from the Animal Department of Anhui Medical University (Hefei, China). Rats were acclimatized and then randomly assigned to four groups: Normal control (NC) group, DN control (DNC) group, DNC plus BBR (100 mg/kg) treatment (DNC+BBR) group and DNC plus enalapril (1 mg/kg) treatment (DNC+Enalapril) group. Fifteen rats were assigned to the NC group, while the other three groups, respectively, to ensure that at least 15 rats survived the entire experiment. After an overnight fast, rats were intraperitoneally administered a single injection of STZ (35 mg/kg) dissolved in a 0.1 mM chilled citrate‐phosphate buffer (pH 4.5). NC rats were injected with citrate–phosphate buffer. The development of hyperglycaemia (diabetes) was confirmed by fasting blood glucose (FBG) estimation (≥11.1 mmol/l) 72 hrs after injection, and only uniformly diabetic rats were included in the DNC, DNC+BBR and DNC+Enalapril groups. NC and DNC rats were given equal volumes of sodium carboxymethyl cellulose (CMC‐Na), while the rest were administered BBR (100 mg/kg/day) and enalapril (1 mg/kg/day) solution intragastrically, for 8 weeks. All experiments were approved by the Ethics Review Committee for Animal Experimentation of the Anhui Medical University.

### Biochemical characteristics analysis

After the experiment, the rats were weighed and placed in metabolic cages for 24‐hr urine collection. Blood samples were collected, and the supernatants were used for the measurement of blood urea nitrogen (BUN), serum creatinine (SCr) and the ratio of urine total protein to urine creatinine (UTP/C) using an automated biochemistry analyzer (Model 7600 Series Automatic Analyzer; Hitachi Corporation, Tokyo, Japan).

### Morphological analysis

Kidney samples were processed using the standard protocol for paraffin embedding. The specimens were sectioned at 4 μm‐thick and stained with periodic acid‐Schiff stain (PAS) prior to examination.

### ELISA assay

Kidney tissue homogenates and cultured media supernatants were collected for PGE_2_ measurement using an ELISA kit following the standard protocol. PGE_2_ levels were normalized to the amount of cellular protein to which the conditioned media were exposed. The cross‐reactivity between PGE_2_ and PGF_2a_ was <0.01%.

### Western blot assay

Kidney tissues and cultured cells were washed with precooling phosphate buffer, and then lysed with lyolysis. Proteins were extracted and subjected to SDS‐PAGE and subsequently transferred to a polyvinylidenfluoride membrane. The membrane was probed with rabbit monoclonal anti‐EP1 antibody (tissue‐derived samples, 1:500; cell‐derived samples, 1:800), anti‐GRK2 antibody (1:500), mouse polyclonal anti‐β‐arrestin2 and anti‐Gαq antibody (1:500), and β‐actin was used as an internal control. After incubation with anti‐rabbit or mouse IgG (1:30,000), immune complexes were detected using the Super Signal Chemiluminescence Kit (Thermo Scientific, Rockford, IL, USA).

### Primary culture of GMCs

Kidneys were aseptically harvested and then stored in precooled Hanks’ buffered salt solution (HBSS; 137 mM NaCl, 5.4 mM KCl, 1.2 mM CaCl_2_, 0.8 mM MgSO_4_, 5.6 mM D‐glucose, 4.1 mM NaHCO_3_, Na_2_HPO_4_·12H_2_O and 15 mM Hepes, pH 7.4). Glomeruli were isolated using the sieving method (100 mesh sieve above and 200 mesh sieve below). The fragments were collected and centrifuged until the purity of the glomeruli exceeded 98%; then, the supernatant was discarded. After digestion, the process was terminated using Dulbecco's modified Eagle's Medium containing 10% foetal bovine serum. The cells were uniformly scattered into culture flasks with antibiotics under appropriate conditions. Cultured cells were identified by hallmarks using a fluorescence immunoassay.

### MTT assay and trypan blue staining

The 3‐(4,5‐dimethylthiazol‐2‐yl)‐2,5‐diphenyl tetrazolium bromide (MTT) assay was used to measure cell proliferation. Briefly, cells were seeded at 10^4^ cells/well in 96‐well plates. The medium was replaced with serum‐free medium for a 24‐hr incubation, and the cells were incubated in the presence or absence of different concentrations of BBR (5, 10, 30, 60, 90, 120 and 240 μM) for 20 and 44 hrs. MTT was added and incubated at 37°C for additional 4 hrs. The medium was carefully removed so as not to disturb the formazan crystals formed. Dimethylsulfoxide was added to each well, and the absorbance of solubilized blue formazan was read at a wavelength of 490 nm using a microplate reader. The optical densities of six wells in each group were measured and normalized to those of the normal group. Meanwhile, cell viability was determined by trypan blue staining to choose the appropriate concentration of BBR for such experiments. The same experiment was used to determine the proper concentration of PGE_2_.

### Desensitization of EP1 receptor

Prostaglandin E_2_ was used to stimulate the desensitization of the EP1 receptor in cultured cells. Briefly, the medium was replaced with serum‐free medium for 24 hrs and then incubated with PGE_2_ at different intervals. Flow cytometry and western blot assay were used to detect the EP1 expression on the cell membrane at different time points (0, 5, 10, 15, 20, 30, 60 and 120 min.). Fluorescent intensities were compared.

### Laser confocal scanning microscopy analysis

Briefly, after treatment with or without BBR, cells were washed with phosphate‐buffered saline, suspended in HBSS and then loaded for 40 min. with 4 μM fluo‐3/AM mixed with Pluronic F127 in the dark. Then, the cells were washed, resuspended and assayed by laser confocal scanning microscopy (LCSM) (Leica TCS‐SP2, Wetzlar, Germany). The fluo‐3 fluorescence intensity was monitored with 488 nm excitation and 526 nm emission wavelengths in cell suspension in the dark.

### Statistical analysis

Data were assessed using SPSS 16.0 (IBM Corporation, Armonk, NY, USA). Significant differences were evaluated using one‐way anova using a post hoc Bonferroni correction (GraphPad Prism 5.0; GraphPad Software, La Jolla, CA, USA). A two‐sided *P* < 0.05 was considered significant. The data are presented as the mean ± S.D. (*n* ≥ 3).

## Results

### Effect of BBR on metabolic parameters

**Figure 1:**
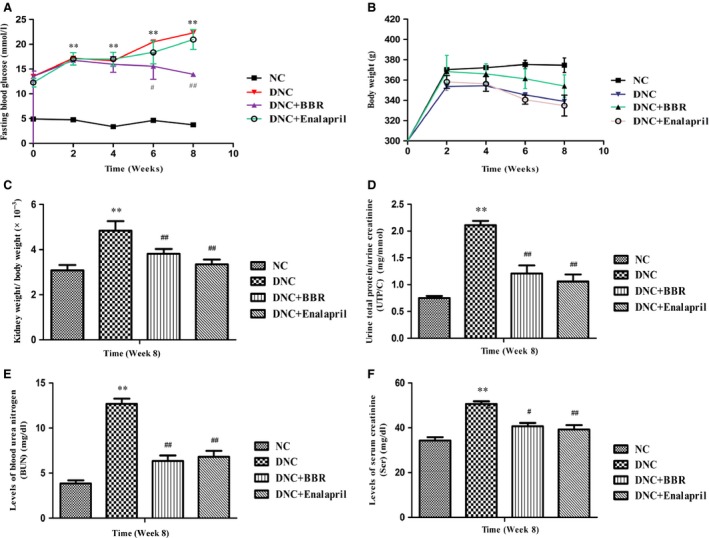
Berberine ameliorated abnormalities of renal function and biochemical markers of DN rats. Fasting blood glucose (**A**), body weight (**B**), ratio of kidney weight to body weight (**C**), ratio of urine total protein to urine creatinine (UTP/C) (**D**), blood urea nitrogen (BUN) (**E**) and serum creatinine (SCr) (**F**) levels were determined. Values are the mean ± S.D., *n* = 15. **P < 0.01 *versus* the NC group; ^#^ *P* < 0.05 and ^#^^#^ *P* < 0.01 compared with the DNC group.

A significant increase in FBG was detected after the induction of diabetes. Administration of BBR had an undistinguishable hypoglycaemic effect in short‐term treatment (0–4 week), while the long‐term treatment (6–8 week) was shown to be satisfactory compared with the DNC group. Enalapril had little impact on reducing the FBG level (Fig. 1A). Diabetic rats had lower body weights, while BBR treatment had no effect on regaining the abnormal weight (Fig. 1B). The ratio of kidney weight to body weight in diabetic rats was significantly increased compared with the NC group (*P* < 0.01), while BBR effectively decreased the ratio (Fig. 1C). The results of enalapril treatment were similar to the results of BBR treatment for both parameters. The levels of UTP/C, BUN and SCr in the DNC group were significantly higher than those of the NC rats. Berberine and enalapril exhibited a remarkable decrease in these indexes (Fig. 1D–F).

### Effect of BBR on renal histopathology

**Figure 2:**
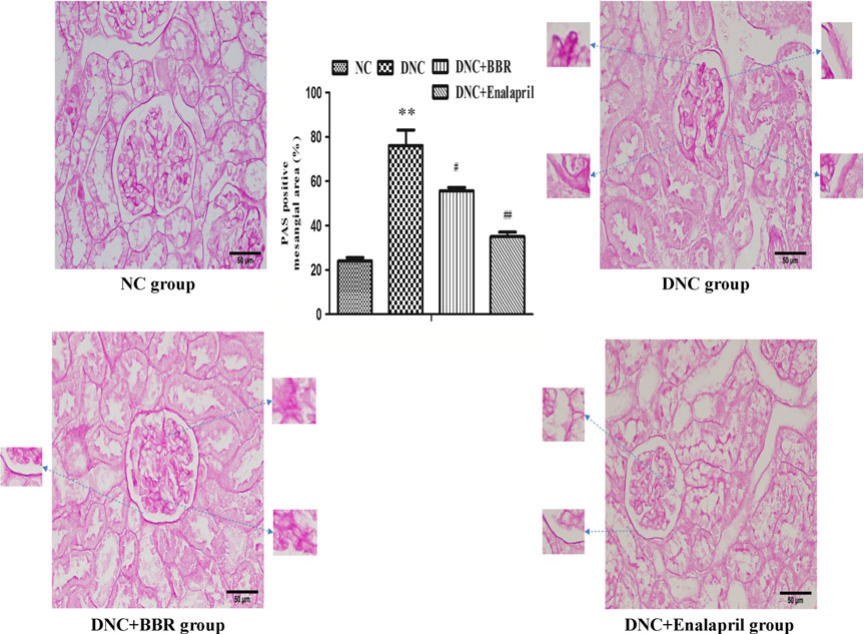
Berberine attenuated pathological changes in DN rats. PAS staining from NC rats and streptozotocin‐induced rats without (DNC) or with berberine (DNC+BBR) and enalapril (DNC+Enalapril) treatment. The bar graph shows the changes of PAS‐positive mesangial area (%). The results are expressed as the mean ± S.D., *n* = 5. **P < 0.01 *versus* NC rats; ^#^ *P* < 0.05 and ^#^^#^ *P* < 0.01 compared with DNC rats.

Untreated rats exhibited marked ECM accumulation, mesangial expansion, glomerular sclerosis and atrophy. When given BBR, pathological changes, such as GBM thickening and ECM accumulation, were scarcely detected, and mesangial expansion was also extremely reduced. However, glomerular atrophy and sclerosis were observed to some extent. Meanwhile, enalapril exhibited a great outcome of improving histological features (Fig. 2).

### Effect of BBR on the level of PGE_2_ in kidney tissue

**Figure 3:**
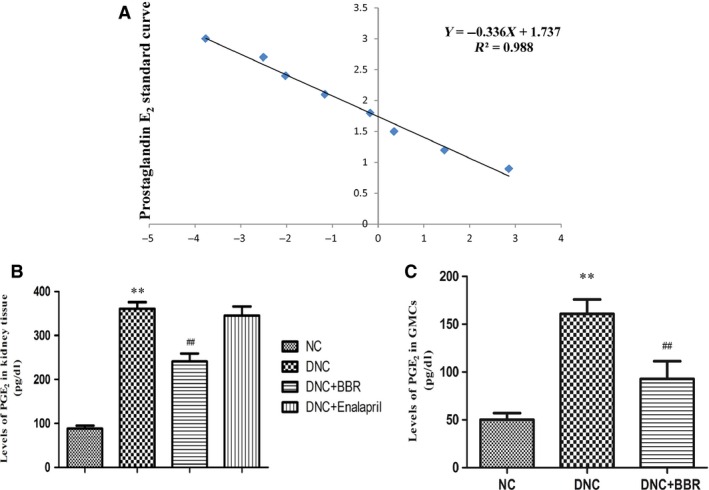
Effect of berberine on the levels of PGE_2_ in DN rat kidneys and cultured GMCs. (**A**) Effect of berberine on the level of PGE_2_ in DN rat kidneys. (**B**) Effect of berberine on the level of PGE_2_ in cultured GMCs. **(C)** The standard curve of PGE_2_ level. The data are expressed as the mean ± S.D., *n* = 5. **P < 0.01 *versus* NC rats; ^#^^#^ *P* < 0.01 compared with DNC rats.

As shown, PGE_2_ secretion was markedly elevated in kidneys of the DNC group by more than fourfold, and such an induction was remarkably blunted by BBR treatment compared with the DNC rats (*P* < 0.05) (Fig. 3B).

### Effect of BBR on the expression of EP1 in kidney tissue

**Figure 4:**
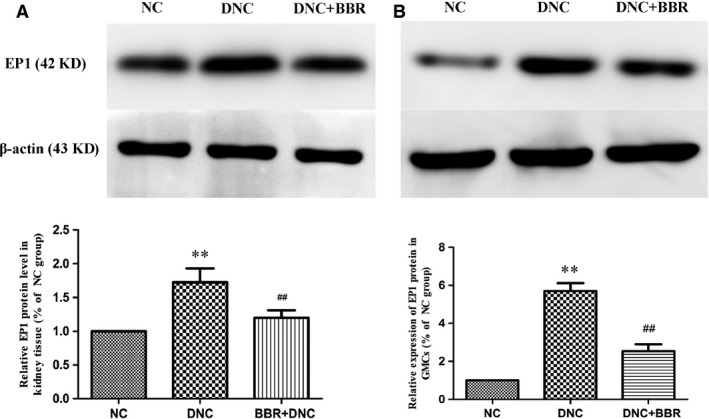
Effect of berberine on the expression of EP1 in DN rat kidneys and cultured GMCs. (**A**) Representative images and quantification of protein by western blot for the EP1 receptor in DN rat kidneys are shown. β‐actin served as an internal control. (**B**) Cells were treated in the presence or absence of berberine (60 μM) for 15 min. EP1 expression was detected by western blotting with anti‐EP1 antibody assay, and the intensity of each band was normalized to β‐actin. Values are the mean ± S.D. of five experiments. **P < 0.01 *versus* NC rats; ^#^^#^ *P* < 0.01 compared with DNC rats.

Western blotting revealed a significant increase in renal EP1 expression in DNC rats (1.7‐fold of NC; *P* < 0.05), whereas protein expression was attenuated by BBR treatment (0.7‐fold of DNC; *P* < 0.05) (Fig. 4A).

### Identification of cultured cells

**Figure 5:**
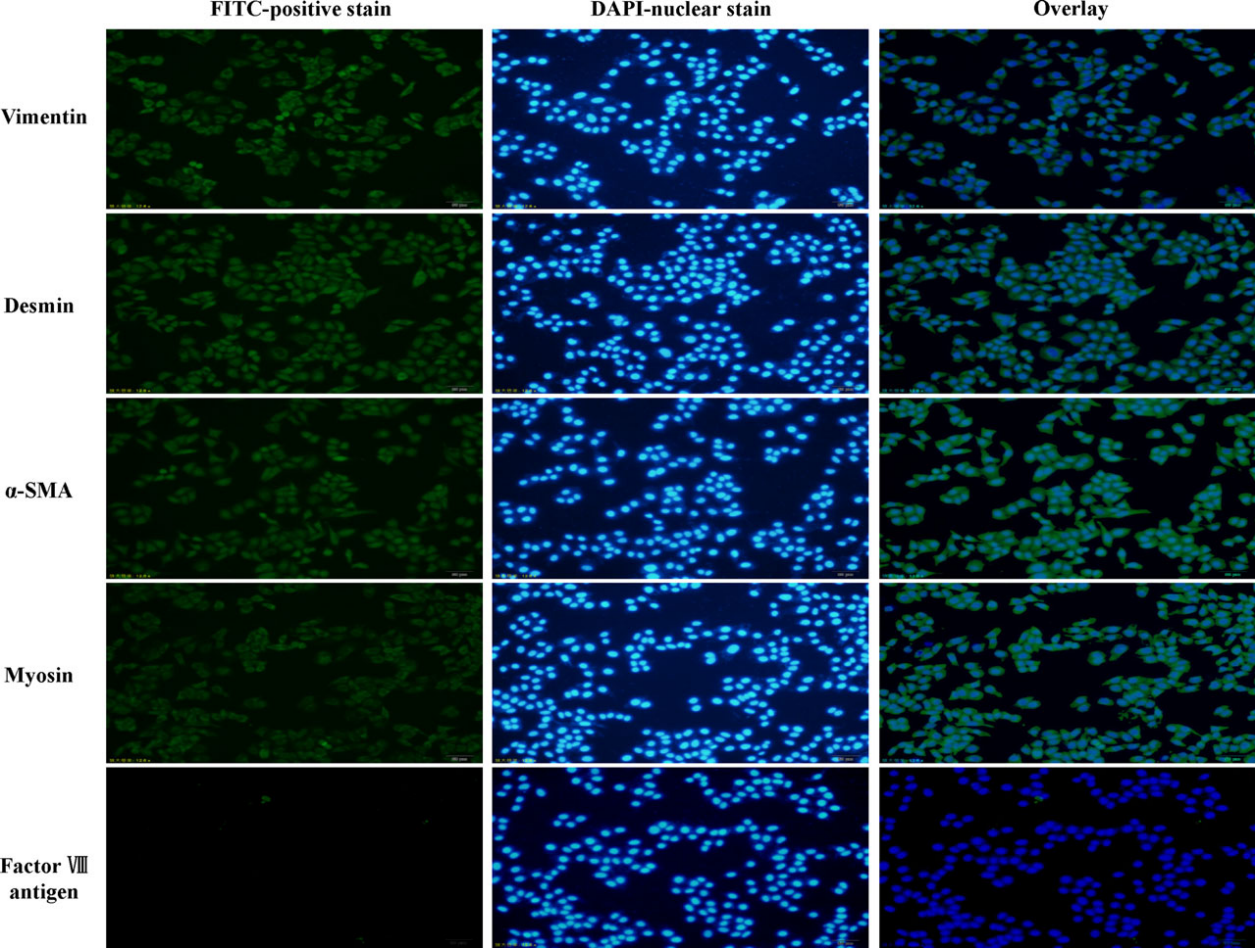
Immunofluorescence photomicrographs of cultured cells. Immunofluorescence was performed on cultured cells using an inverted microscope (scale bar: 50 μm). The results showed that the cultured cells had positive staining for vimentin, desmin, α‐smooth muscle actin (α‐SMA) and myosin but negative staining for factor VIII antigen.

Fluorescence immunoassay using an inverted microscope showed that the cultured cells had positive staining for of vimentin, desmin, α‐smooth muscle actin and myosin but negative staining for factor VIII antigen. The results confirmed that the cultured cells are GMCs (Fig. 5).

### Effect of BBR on proliferation of GMCs

**Figure 6:**
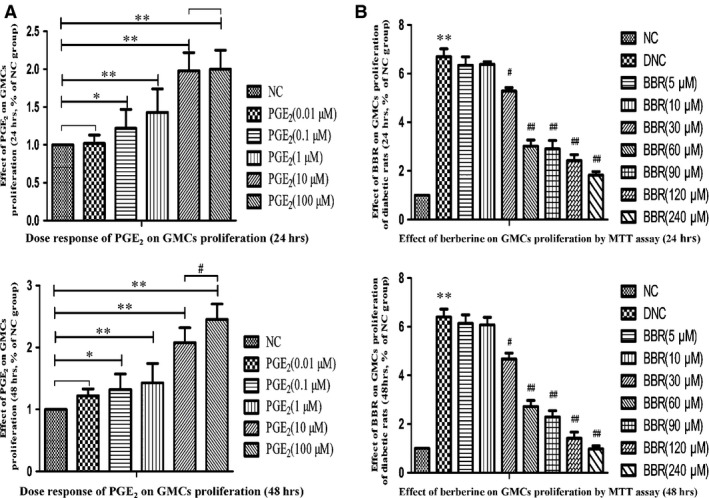
Dose response of PGE_2_ and berberine on the proliferation of GMCs by MTT assay. (**A**) Dose response of PGE_2_ on the proliferation of GMCs by MTT assay. (**B**) Effect of berberine on the proliferation of GMCs by MTT assay. Values are the mean ± S.D of four experiments. *P < 0.05 and **P < 0.01 *versus* the NC group; ^#^ *P* < 0.05 and ^#^^#^ *P* < 0.01 compared with the DNC group.

An MTT assay was used to assess the impacts of BBR and PGE_2_ on the proliferation of GMCs. Interestingly, it was noted that administration of BBR for 24 or 48 hrs significantly suppressed the proliferation of GMCs, with a minimum concentration of 30 μM (*P* < 0.05). Trypan blue staining showed that cell mortality was less than 15% at the peak concentration of 90 μM, while cell mortality was 5% at a concentration of 60 μM (Table 1). Based on Figure 6B, 60 μM was considered a therapeutic concentration, and 10 μM was the optimum concentration of PGE_2_ according to Figure 6A.

**Table 1:** Trypan blue dyeing determined the cells’ vigor

BBR	Cell mortality (%)	
	BBR (24 hrs)	BBR (48 hrs)
5 (μM)	2.0 ± 0.10	2.5 ± 0.11
10 (μM)	2.0 ± 0.12	3.1 ± 0.10
30 (μM)	3.1 ± 0.28	3.2 ± 0.23
60 (μM)	4.6 ± 0.25	4.9 ± 0.21
90 (μM)	9.6 ± 0.10	16.0 ± 0.41
120 (μM)	15 ± 0.14	19.5 ± 0.25
240 (μM)	17.2 ± 0.21	16.3 ± 0.32

Cells were treated with different concentrations of berberine and the inhibitory ratio was calculated by the following formula: Cell mortality (%) = (the dead cell number/the total number) × 100%. Values are mean ± S.D., *n* = 3.

### Desensitization of EP1 receptor in GMCs

**Figure 7:**
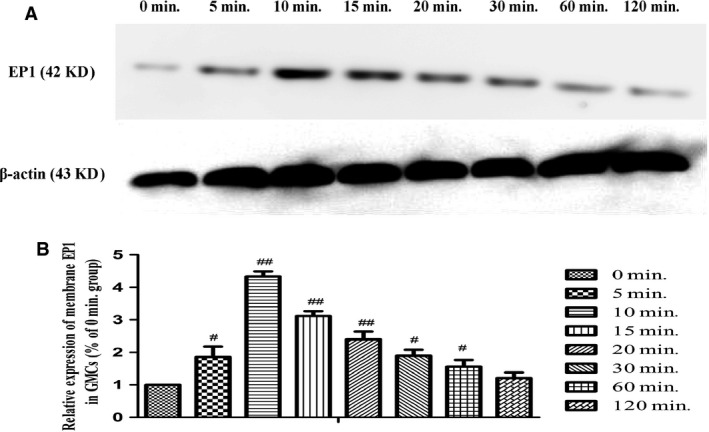
Membrane expression of the EP1 receptor in GMCs stimulated by PGE_2_ at multiple time‐points. (**A**) Representative images of western blot for membrane EP1 expression are shown. β‐actin served as the loading control. (**B**) Quantification of western blot for EP1 expression in GMCs stimulated by PGE_2_ at multiple time‐points. Data are the mean ± S.D. of at least triple experiments. ^#^ *P* < 0.05 and ^#^^#^ *P* < 0.01 compared with the 0 min. group.

**Figure 8:**
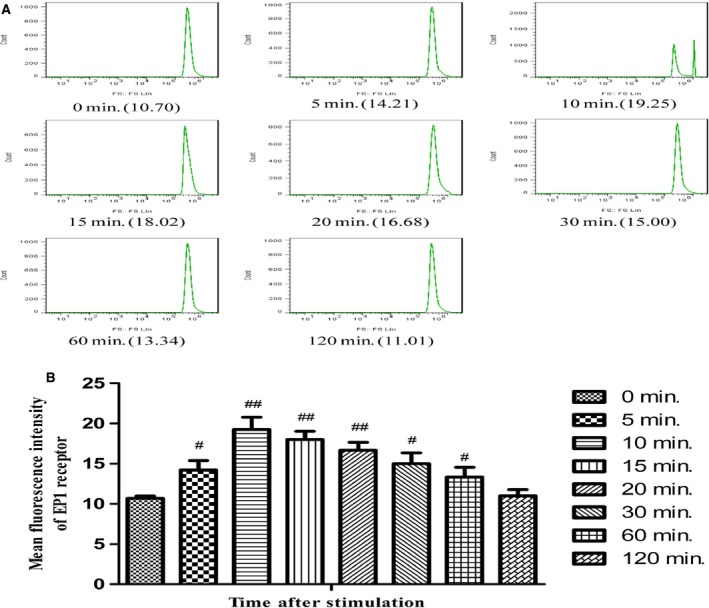
Membrane expression of the EP1 receptor in GMCs stimulated by PGE_2_ at multiple time points. (**A**) Images of flow cytometry for membrane EP1 expression are shown. (**B**) Quantification of flow cytometry for EP1 expression in GMCs stimulated by PGE_2_ at multiple time points. Data are the mean ± S.D. of at least five experiments. ^#^ *P* < 0.05 and ^#^^#^ *P* < 0.01 compared with the 0 min. group.

After stimulation by PGE_2_, membrane EP1 expression in GMCs was gradually increased for 10 min. and then continuously decreased. Membrane EP1 expression was almost flat from that of the control level at 0 min. (Fig. 7). Western blotting showed that membrane EP1 expression reached a peak at 10 min. in response to PGE_2_ stimulation, and then flow cytometry revealed that membrane EP1 expression decreased to near control level at 0 min. (Fig. 8).

### Changes of cytoplasm GRK2 and membrane β‐arrestin2

**Figure 9:**
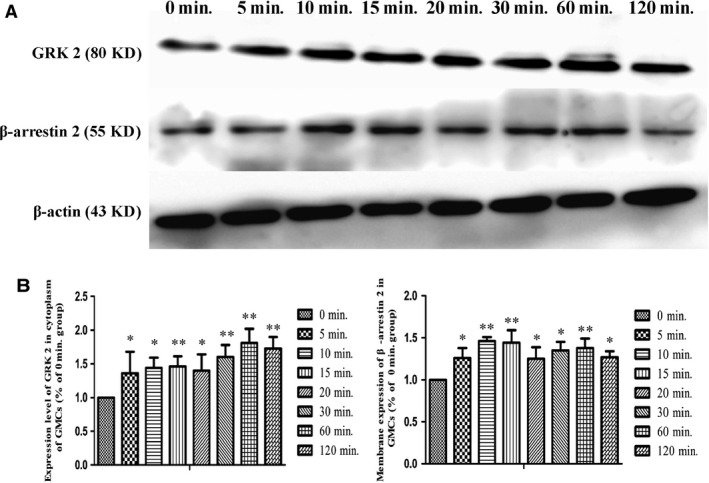
Changes in cytoplasmic GRK2 and membrane β‐arrestin2 expression levels in GMCs at multiple time points. (**A**) Representative images of western‐blot for cytoplasm GRK2 and membrane β‐arrestin2 expression are shown. (**B**) The intensity of each band with anti GRK2 and anti β‐arrestin2 antibodies were quantified and normalized to that of β‐actin. Values are the mean ± S.D. of five experiments. *P < 0.05 and **P < 0.01 *versus* the 0 min. group.

The expression level of GRK2 in the cytoplasm was clearly enhanced after 10 min. with PGE_2_ stimulation compared with 0 min. (*P* < 0.01). Interestingly, membrane β‐arrestin2 continuously increased after 10 min. under PGE_2_ induction, and it remained flat close to that level (Fig. 9).

### Effect of BBR on the level of PGE_2_ in GMCs

The data indicated that the level of PGE_2_ in GMCs of the DNC group was significantly elevated and was higher than that of the NC group. Berberine could effectively inhibit the increased PGE_2_ level (*P* < 0.01) compared with that of the DNC group (Fig. 3C).

### Effect of BBR on expression of EP1 and Gαq in GMCs

**Figure 10:**
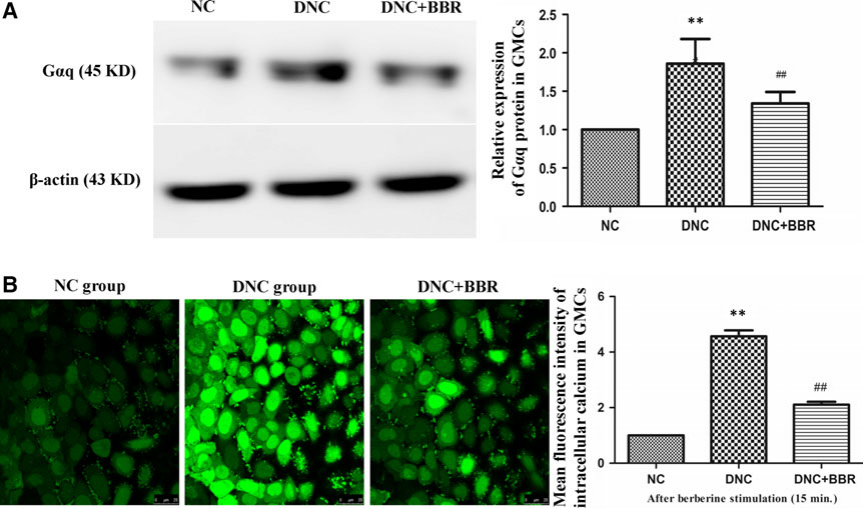
Effect of berberine on the expression of Gαq and the concentration of [Ca^2+^]i in GMCs. (**A**) Cells were treated with or without berberine (60 μM) for 15 min. Gαq expression was detected by western blotting with an anti‐Gαq antibody, and the intensity of each band was normalized to β‐actin. (**B**) The distribution of [Ca^2+^]i in GMCs at 15 min. after berberine treatment in the different groups (Scale bar: ×25 μm) and the fluorescence intensity of each group for [Ca^2+^]i. Experiments were performed at least five times with similar results. **P < 0.01 *versus* the NC group; ^#^^#^ *P* < 0.01 compared with the DNC group.

Compared with the NC group, increased expression of EP1 in GMCs with DN was observed, and increased expression of the Gαq protein was also observed. Berberine could apparently inhibit the expression levels of EP1 (Fig. 4B) and Gαq (Fig. 10A), as observed in the kidneys.

### Effect of BBR on the concentration of Ca^2+^ in GMCs

The results showed that, in the DNC group, the fluorescence intensity of cytosolic calcium increased and was significantly higher than that of the NC group, which meant that the concentration of free calcium in the cells of the DNC group was markedly higher than that in the NC group. Meanwhile, BBR‐treated cells showed markedly decreased fluorescence intensity in GMCs, which indicated that BBR could significantly decrease the level of cytoplasmic calcium (Fig. 10B).

## Discussion

Diabetic nephropathy is characterized by progressive renal failure, which is mainly caused by GMC proliferation and augmented deposition of ECM proteins at the glomerular level, thus inducing mesangial expansion, glomerulosclerosis and atrophy 19. During the process, abnormal changes of renal function were mainly manifested as significant increases in kidney indexes, such as SCr, BUN and UTP/C, from a macroscopic viewpoint 20. Current interventions for DN, namely, control of blood glucose, hypertension and blockade of the renin–angiotensin system, can hinder but not prevent the progressive loss of renal function that frequently accompanies DN 21. Accordingly, the identification of new anti‐DN drugs continues to be pursued. In this study, we explored the renoprotective roles of BBR and found that ratios of KW/BW and UTP/C as well as levels of SCr and BUN were significantly increased compared with the NC group, which indicated that the model was successfully established and glomerular injury and renal dysfunction emerged. Compared with the DNC group, BBR significantly ameliorated abnormal renal function beginning at the sixth week, which suggested that BBR exerted its renoprotective effect by improving functional parameters, but prolonged treatment is needed. In addition, BBR improved pathological alterations such as ECM accumulation, mesangial expansion, glomerulosclerosis and glomerular atrophy based on histological changes in the kidneys of DN rats. These findings supported the view that BBR can attenuate the progression of DN. Mesangial hypercellularity or cell proliferation preceded an increase in ECM proteins, subsequent ECM accumulation, and importantly, mesangial hypertrophy, which were associated with eventual glomerulosclerosis and atrophy 22. Of course, there is an increasing awareness and consensus that the abnormal growth and activity of GMCs plays an important role in the pathophysiology of the early stages of DN. In addition, GMCs are considered specialized smooth muscle cells, which play pivotal roles in the regulation of glomerular haemodynamics. In glomeruli, GMCs formed a tree‐like network, branching from the hilar site to the glomerular capillary loops and connecting with each other 23. According to one report, BBR has multiple pharmacological activities, which indicate its wide clinical and research value as a therapeutic anti‐DN drug 24. According to the pathological analysis, BBR reduced the proliferation of GMCs in the DN state, thus reducing ECM accumulation and ameliorating glomerular sclerosis and atrophy. What is the mechanism of BBR when targeting GMCs in DN rats? In cellular activities, signalling transduction is a critical element and the foundation of the pathophysiological process, which is quite complex and has not been completely interpreted at present. Research demonstrated that PGE_2_ is an inflammatory mediator that has pleiotropic effects on signalling cascades *via* specific GPCRs, the seven transmembrane proteins that form the largest single family of integral membrane receptors. In particular, excessive PGE_2_ has been shown to be associated with an increased glomerular filtration rate in the early stage of DN and with subsequent glomerular hypertrophy, proteinuria and renal injury 25. Further analysis using an ELISA assay showed that the production of PGE_2_ in both kidney tissue and the supernatant of cultured GMCs were significantly elevated. On the basis of molecular and pharmacological research, the corresponding GPCRs, namely, the ‘EP receptors’, are now divided into four subtypes, EP1, EP2, EP3 and EP4, which are similar in structure, but differ in terms of tissue distribution. E‐prostanoid receptors transduce information provided by extracellular stimuli into intracellular second messengers *via* coupling to heterotrimeric G proteins and the subsequent regulation of a diverse variety of effector systems after binding to ligand 26. In this study, the expression of the EP1 receptor increased significantly both in glomeruli and cultured GMCs, which indicated that the PGE_2_/EP1 signalling pathway was activated and may transmit signals that regulate the activities of GMCs in the DN state. In the transduction process, the reversible phosphorylation of G protein kinase (GRK) played critical roles in signal identification and transfer in cells. There are seven known GRKs, and GRK2 is the most widely expressed. GRKs, however, preferentially phosphorylate receptors that are in the agonist‐occupied conformation. Under such circumstance, homologous desensitization or internalization of GPCR was mediated by phosphorylation of the receptor and subsequent binding of β‐arrestin, which were concentrated inside of the cell membrane along with GRKs 27. In addition, the recently emerging and constantly expanding field of heptahelical receptor GRK‐β‐arrestin‐dependent signalling also offered several exciting new opportunities for therapeutic intervention in many diseases. Despite still being in its infancy, as far as identification of specific physiological and pathophysiological effects is concerned, there is a huge potential for exploiting DN for therapeutic purposes. Flow cytometry was used to determine the membrane levels of EP1 expression on GMCs after stimulation by PGE_2_ at multiple time points, and the results of western blotting showed that the membrane level of EP1 expression gradually increased up to the peak at 10 min., and then decreased to the control level. At this timepoint, the expression level of GRK2 in the cytoplasm was measured, and the results showed that there was no significant change in GRK2 level in the cytoplasm from 0 to 10 min., and afterward the expression level of GRK2 in the cytoplasm increased significantly. However, the membrane expression of β‐arrestin2 continuously increased after 10 min. The fluorescence intensity of GMC stimulated by PGE_2_ was enhanced in response to PGE_2_ activation. At approximately 10 min., the fluorescence intensity of EP1 on the GMC membrane was markedly increased, and then it gradually decreased, indicating the internalization of EP1. Such expression changes of GRK2 and β‐arrestin2 led us to hypothesize that the internalization of EP1 was modulated by GRK2 and β‐arrestin2. In the subsequent experiments, BBR was applied to the cultured cells after stimulation with PGE_2_ for 15 min. Western bloting was used to detect alterations of the EP1 protein and the corresponding G protein, *i.e*., Gαq, and the results showed that the expression levels of EP1 and the Gαq protein were markedly increased compared with the NC group, while BBR effectively attenuated the over‐expression of EP1 and Gαq. Based on the above findings, we hypothesized that BBR altered the expression of EP1 and then decreased the level of Gαq in the intracellular communication, eventually mediating the PGE_2_‐EP1‐Gαq signalling pathway by regulating the desensitization of the EP1 receptor. On the basis of the signalling pathway analysis, the concentrations and changes of cytosolic‐free calcium ions in the DN condition were examined by LCSM. The results showed that intracellular free calcium fluorescent intensity was markedly elevated due to PGE_2_ stimulation in GMCs. By contrast, fluorescent density in the BBR‐treated group was markedly decreased compared with the DNC group, which indicated that BBR may effectively regulate the activities of GMCs by reducing the intracellular concentration of free calcium ion. Intracellular Ca^2+^ activity, or intracellular Ca^2+^ concentration ([Ca^2+^]i), is an important biological signal in the control of protein secretion, contraction, development and apoptosis in a wide variety of cells. Many cellular functions critically depend on [Ca^2+^]i and a sustained elevation in [Ca^2+^]i is capable of activating multiple potentially harmful cellular processes, including phenotypic change, dysregulated cell proliferation, cell injury, apoptosis, and death 28. Like vascular smooth muscle cells, many different functions of GMCs were controlled by [Ca^2+^]i. Under such conditions, GMCs changed their phenotype and became proliferative and matrix expanding subsequently, they promoted ECM accumulation, mesangial hypertrophy and even glomerulosclerosis and ultimately accelerated the progression of DN 29. Meanwhile, one study reported that PGE_2_/EP1 signals mediated cytosolic Ca^2+^ channels 30. Therefore, it would be reasonable to conclude that BBR may affect the abnormal proliferation, contraction and cytokine and protein biosynthesis of GMCs by regulating PGE_2_‐EP1‐Gαq‐Ca^2+^ signalling transduction to ameliorate the above symptoms. Whether targeting the PGE_2_‐EP1‐Gαq‐Ca^2+^ signalling pathway represents a new therapeutic option for diabetic patients remains to be established in further studies. To date, the pathogenesis of DN has not been completely clarified, and the exact mechanisms of BBR treatment are not well understood. More in‐depth analysis should unravel the profound mystery of and explore the application of BBR. We next plan to test these possibilities by knocking‐out or silencing the EP1 gene, and then we can elaborate on the exact renoprotective mechanism of BBR at the gene levels by determining the mRNA expression of EP1 and the corresponding molecules.

## Conclusions

In summary, BBR not only attenuates DN by ameliorating changes in renal pathophysiology but also by suppressing the proliferation of GMCs through the PGE_2_‐EP1‐Gαq‐Ca^2+^ signalling pathway. The data presented here support the concept that EP1 may be an important therapeutic target for DN treatment and other kidney diseases related to the PGE_2_‐EP1 signalling pathway.

The importance of BBR in treating DN leads us to pursue a more detailed analysis, while a better understanding of the mechanisms and physicochemical properties will provide sufficient theoretical support for us to seek the possible therapeutic targets and promote the clinical application of BBR in DN 31. However, to date, there is no report detailing the effects of BBR on diabetic patients, indicating the enormous potential application in treating diabetic kidney disease in patients.

## Conflicts of interest

The authors confirm that there are no conflicts of interest.

## Author contribution

WJ Ni and HH Ding performed the research; WJ Ni, LQ Tang, HH Ding, H Zhou and YY Qiu designed the research study; WJ Ni, LQ Tang, HH Ding, H Zhou and YY Qiu contributed essential reagents or tools; WJ Ni and LQ Tang analysed the data; WJ Ni and LQ Tang wrote the paper.

## References

[1] American Diabetes Association . Executive summary: standards of medical care in diabetes–2014. Diabetes Care. 2014; 37: S5–13. 24357214 10.2337/dc14-S005

[2] Sidaway P . Diabetic nephropathy: heparanase mediates renal injury. Nat Rev Nephrol. 2014; 10: 483. 10.1038/nrneph.2014.134 25072118

[3] Blantz RC . Phenotypic characteristics of diabetic kidney involvement. Kidney Int. 2014; 86: 7–9. 24978373 10.1038/ki.2013.552 PMC4076684

[4] Peng J , Li X , Zhang D , *et al* Hyperglycemia, p53, and mitochondrial pathway of apoptosis are involved in the susceptibility of diabetic models to ischemic acute kidney injury. Kidney Int. 2015; 87: 137–50. 24963915 10.1038/ki.2014.226 PMC4276728

[5] Roscioni SS , Heerspink HJ , de Zeeuw D . The effect of RAAS blockade on the progression of diabetic nephropathy. Nat Rev Nephrol. 2014; 10: 77–87. 24296623 10.1038/nrneph.2013.251

[6] Ni WJ , Tang LQ , Wei W . Research progress in signalling pathway in diabetic nephropathy. Diabetes Metab Res Rev. 2015; 31: 221–33. 24898554 10.1002/dmrr.2568

[7] Shindou H , Hishikawa D , Harayama T , *et al* Recent progress on acyl CoA: lysophospholipid acyltransferase research. J Lipid Res. 2009; 50: S46–51. 18931347 10.1194/jlr.R800035-JLR200 PMC2674719

[8] Capra V , Back M , Barbieri SS , *et al* Eicosanoids and their drugs in cardiovascular diseases: focus on atherosclerosis and stroke. Med Res Rev. 2013; 33: 364–438. 22434418 10.1002/med.21251

[9] Yang Y , Tang LQ , Wei W . Prostanoids receptors signaling in different diseases/cancers progression. J Recept Signal Transduct Res. 2013; 33: 14–27. 23327583 10.3109/10799893.2012.752003

[10] Eskildsen MP , Hansen PB , Stubbe J , *et al* Prostaglandin I2 and prostaglandin E2 modulate human intrarenal artery contractility through prostaglandin E2‐EP4, prostacyclin‐IP, and thromboxane A2‐TP receptors. Hypertension. 2014; 64: 551–6. 24914192 10.1161/HYPERTENSIONAHA.113.03051

[11] Omori K , Kida T , Hori M , *et al* Multiple roles of the PGE2 ‐EP receptor signal in vascular permeability. Br J Pharmacol. 2014; 171: 4879–89. 24923772 10.1111/bph.12815 PMC4294111

[12] Price SR , Klein JD . Cyclooxygenase‐2 in the kidney: good, BAD, or both? Kidney Int. 2011; 80: 905–7. 21997504 10.1038/ki.2011.263 PMC3664549

[13] Stanley C , O'Sullivan SE . Vascular targets for cannabinoids: animal and human studies. Br J Pharmacol. 2014; 171: 1361–78. 24329566 10.1111/bph.12560 PMC3954478

[14] Kennedy CR , Xiong H , Rahal S , *et al* Urine concentrating defect in prostaglandin EP1‐deficient mice. Am J Physiol Renal Physiol. 2007; 292: F868–75. 16885154 10.1152/ajprenal.00183.2005

[15] Faour WH , Thibodeau JF , Kennedy CR . Mechanical stretch and prostaglandin E2 modulate critical signaling pathways in mouse podocytes. Cell Signal. 2010; 22: 1222–30. 20362052 10.1016/j.cellsig.2010.03.014

[16] Zhao HL , Sui Y , Qiao CF , *et al* Sustained antidiabetic effects of a berberine‐containing Chinese herbal medicine through regulation of hepatic gene expression. Diabetes. 2012; 61: 933–43. 22396199 10.2337/db11-1164 PMC3314348

[17] Ni WJ , Ding HH , Tang LQ . Berberine as a promising anti‐diabetic nephropathy drug: an analysis of its effects and mechanisms. Eur J Pharmacol. 2015; 760: 103–12. 25912800 10.1016/j.ejphar.2015.04.017

[18] Tang LQ , Wang FL , Zhu LN , *et al* Berberine ameliorates renal injury by regulating G proteins‐AC‐ cAMP signaling in diabetic rats with nephropathy. Mol Biol Rep. 2013; 40: 3913–23. 23266672 10.1007/s11033-012-2468-0

[19] Allison SJ . Diabetic nephropathy: HIF activation in prevention of diabetic nephropathy. Nat Rev Nephrol. 2014; 10: 612. 25247333 10.1038/nrneph.2014.177

[20] Haase VH . A breath of fresh air for diabetic nephropathy. J Am Soc Nephrol. 2015; 26: 239–41. 25183808 10.1681/ASN.2014080754 PMC4310668

[21] Fineberg D , Jandeleit‐Dahm KA , Cooper ME . Diabetic nephropathy: diagnosis and treatment. Nat Rev Endocrinol. 2013; 9: 713–23. 24100266 10.1038/nrendo.2013.184

[22] Ni WJ , Ding HH , Zhou H , *et al* Renoprotective effects of berberine through regulation of the MMPs/TIMPs system in streptozocin‐induced diabetic nephropathy in rats. Eur J Pharmacol. 2015; 764: 448–56. 26192633 10.1016/j.ejphar.2015.07.040

[23] Tsurumi H , Harita Y , Kurihara H , *et al* Epithelial protein lost in neoplasm modulates platelet‐derived growth factor‐mediated adhesion and motility of mesangial cells. Kidney Int. 2014; 86: 548–57. 24694988 10.1038/ki.2014.85

[24] Liu L , Liu J , Gao Y , *et al* Uncoupling protein‐2 mediates the protective action of berberine against oxidative stress in rat insulinoma INS‐1E cells and in diabetic mouse islets. Br J Pharmacol. 2014; 171: 3246–54. 24588674 10.1111/bph.12666 PMC4080978

[25] Ranganathan PV , Jayakumar C , Mohamed R , *et al* Netrin‐1 regulates the inflammatory response of neutrophils and macrophages, and suppresses ischemic acute kidney injury by inhibiting COX‐2‐mediated PGE2 production. Kidney Int. 2013; 83: 1087–98. 23447066 10.1038/ki.2012.423 PMC3672333

[26] Wang F , Lu X , Peng K , *et al* Prostaglandin E‐prostanoid4 receptor mediates angiotensin II‐induced (pro)renin receptor expression in the rat renal medulla. Hypertension. 2014; 64: 369–77. 24866147 10.1161/HYPERTENSIONAHA.114.03654 PMC4445967

[27] Evron T , Daigle TL , Caron MG . GRK2: multiple roles beyond G protein‐coupled receptor desensitization. Trends Pharmacol Sci. 2012; 33: 154–64. 22277298 10.1016/j.tips.2011.12.003 PMC3294176

[28] Kane DV , Pedi L , Long SB . Structure and insights into the function of a Ca(2+)‐activated Cl(‐) channel. Nature. 2014; 516: 213–8. 25337878 10.1038/nature13913 PMC4454446

[29] Li L , Trifunovic A , Kohler M , *et al* Defects in beta‐cell Ca^2+^ dynamics in age‐induced diabetes. Diabetes. 2014; 63: 4100–14. 24985350 10.2337/db13-1855

[30] Rodriguez‐Lagunas MJ , Martin‐Venegas R , Moreno JJ , *et al* PGE2 promotes Ca^2+^‐mediated epithelial barrier disruption through EP1 and EP4 receptors in Caco‐2 cell monolayers. Am J Physiol Cell Physiol. 2010; 299: C324–34. 20484658 10.1152/ajpcell.00397.2009

[31] Bethel MA , Xu W , Theodorakis MJ . Pharmacological interventions for preventing or delaying onset of type 2 diabetes mellitus. Diabetes Obes Metab. 2015; 17: 231–44. 25312701 10.1111/dom.12401

